# Entropy production and mixed convection within trapezoidal cavity having nanofluids and localised solid cylinder

**DOI:** 10.1038/s41598-021-94238-w

**Published:** 2021-07-19

**Authors:** Muhamad S. Ishak, Ammar I. Alsabery, Ishak Hashim, Ali J. Chamkha

**Affiliations:** 1 Department of Mathematical Sciences, Faculty of Science and Technology, Universiti Kebangsaan Malaysia, 43600 UKM Bangi, Selangor Malaysia; 2 Department of Engineering, College of Foundation and Diploma Studies, Universiti Tenaga Nasional, 43000 Kajang, Selangor Malaysia; 3 Refrigeration and Air-conditioning Technical Engineering Department, College of Technical Engineering, The Islamic University, Najaf, Iraq; 4 Faculty of Engineering, Kuwait College of Science and Technology, Doha District, 35001 Kuwait; 5 Center of Excellence in Desalination Technology, King Abdulaziz University, P.O. Box 80200, Jeddah, 21589 Saudi Arabia

**Keywords:** Energy science and technology, Engineering, Mathematics and computing, Nanoscience and technology

## Abstract

The entropy production and mixed convection within a trapezoidal nanofluid-filled cavity having a localised solid cylinder is numerically examined using the finite element technique. The top horizontal surface moving at a uniform velocity is kept at a cold temperature, while the bottom horizontal surface is thermally activated. The remaining surfaces are maintained adiabatic. Water-based nanofluids ($\text {Al}_2\text {O}_3$ nanoparticles) are used in this study, and the Boussinesq approximation applies. The influence of the Reynolds number, Richardson number, nanoparticles volume fraction, dimensionless radius and location of the solid cylinder on the streamlines, isotherms and isentropic are examined. The results show that the solid cylinder’s size and location are significant control parameters for optimising the heat transfer and the Bejan number inside the trapezoidal cavity. Furthermore, the maximum average Nusselt numbers are obtained for high *R* values, where the average Nusselt number is increased by 30% when *R* is raised from 0 to 0.25.

## Introduction

Mixed convective heat transfer inside cavities acts as an extensive rule in various engineering applications such as solar panels, material processors, solar ponds, heat exchangers and many more. Several researchers have investigated the combination of the shear effect and buoyancy force. For instance, mixed convection in square or rectangle cavities have been investigated in several studies by Roy et al.^1^, Abbasian Arani et al.^2^, Khorasanizadeh et al.^3^, Sebdani et al.^4^ and Nayak et al.^5^. Additionally, some researchers have performed numerical investigations on mixed convection inside trapezoidal enclosures. Arefmanesh et al.^6^ concluded that the nanofluid’s effective thermal conductivity for a variable-property form had a significant performance toward the entropy production and heat transfer within the employed enclosure. Bhattacharya et al.^7^ stated that the non-isothermal bottom wall plays an important role in multiple steady elements into either natural convection controlled regime or mixed convection regime for convection dominated heat transport regime. Kareem et al.^8^ investigated mixed convection in a trapezoidal cavity using different types of nanofluid without an inlet opening site. The results indicated that a wide range of nanofluids had a higher Nusselt number than pure water. Furthermore, the Nusselt number increased as the volume fraction increased, and the rotational angle declined as the measurement of the nanoparticles increased. Selimefendigil and Öztop^9^ investigated mixed convection heat transfer into a lid-driven trapezoidal cavity filled with nanofluid and having an inclined magnetic field. The results revealed that the Richardson number, the magnetic field force, and nanoparticle volume fraction were enhanced, while a disparity was observed within the average Nusselt number rises concerning systems by various electrical conductivity criteria. Aghaei et al.^10^ examined the impacts of the magnetic field on entropy production and heat transfer of Cu-water nanofluid mixed convection in a trapezoidal cavity. The authors showed that the convection of nanofluids and the intensity of the flow decreased, while the flow-oriented across natural convection and lastly toward pure conduction by introducing and enhancing the magnetic field. In contrast, the effects of entropy production due to friction were insignificant, and the entire entropy production was mainly because of the irreversibility associated with heat transfer. The average Nusselt number grows with the developing Reynolds number for all Darcy numbers, aspect ratios, and nanoparticle volume fractions^11^. The influence of the cavity’s tilt angle on mixed convection and heat transfer within two separate lid-driven trapezoidal enclosures was previously investigated by Hasib et al.^12^. It did note that the features of heat transfer and fluid flow inside the cavities were entirely conditional on Reynolds and Grashof numbers. Chamkha and Ismael^13^ explored the mixed convection of the right heat-inclined sidewall within a lid-driven nanofluid-filled trapezoidal cavity with the impact of the uniform magnetic field. The authors showed that the Nusselt number’s action varies from the Richardson number associated with the lid’s direction. The numerical outcomes of the heat transfer into mixed convection in a lid-driven flow inside a trapezoidal cavity with a steady magnetic field were examined by Javed et al.^14^. The findings revealed that the effects of the moving lid became negligible for $Ra=10^5$, although the increasing Rayleigh number led to more excellent streamline circulation and dominant convection effects within the enclosure. While in another investigation, the results indicated that the average Nusselt number decreased as the Lewis number increased, while the total entropy production increased^15^. Al-Rashed et al.^16^ examined mixed convection and entropy generation in a cubical heated up by a central isothermal block. Natural convection heat transfer and total entropy generation of nanofluid in a square cavity in presence of a refrigerant rigid body was considered by Rahimi et al.^17^. Recently, Rashidi et al.^18^ investigated a three-dimensional free convection and entropy generation of $\text {Al}_2\text {O3}$ water-based nanofluid in a cylindrical cavity. Natural convection and entropy generation in an air-filled cubical cavity with active lateral walls was studied by Alnaqi et al.^19^.

Several studies have been performed using either the base fluid or nanofluid or the combination of both fluids. For instance, Alsabery et al.^20^ studied natural convective heat transfer in a nanofluid filled-non-Darcian porous and wavy-walled domain under the local thermal non-equilibrium condition. Islam et al.^21^ investigated the MHD convection flow within a prism shape cavity filled with Cu-$\hbox {H}_2$O nanofluid. Numerical simulations of a hybrid nanofuid flow through a permeable curved enclosure was given by Shah et al.^22^. Gibanov et al.^23^ investigated the conjugate mixed convection and entropy production of nanofluid (alumina-water) in a lid-driven cavity with a solid bottom surface. It was shown that the bottom wall thickness and volume fraction of nanoparticles perform an important function in enhancing heat transfer. Astanina et al.^24^ examined the mixed convection of nanofluids (alumina-water) into a lid-driven cavity containing two porous layers with different thermal properties, permeability, and porosity on the bottom wall. The authors discovered that the extension of nanoparticles contributed to the enhancement of heat transfer in natural convection, while the addition in the nanoparticles volume fraction led toward an apparent decline in heat transfer for mixed and forced convection. Mehmood et al.^25^ investigated the mixed convection within nanofluid-filled (alumina-water) lid-driven square cavity containing square blockage and magnetic field. It was observed that as the nanoparticle volume fraction improved, the average Nusselt number and internal energy were increased. Their findings also showed that the rise in the magnetic field directed to reduce the average Nusselt number, and average entropy generation was due to heat transfer. Goodarzi et al.^26^ investigated the two-phase mixture rule laminar and turbulent mixed convective heat transfer of water-Cu nanofluids in a shallow rectangular cavity. By increasing the volume fraction of nanoparticles, the coefficient of convective heat transfer increased together with the Nusselt number for specific Grashof and Richardson numbers. The laminar mixed convection flow inside lid (single and double) square cavities filled with nanofluids (alumina-water) was also investigated numerically by Chamkha and Abu-Nada^27^. Due to nanoparticles’ existence, it was observed that notable heat transfer enhancement did accomplish, which was strengthened by growing the nanoparticle volume fraction on moderate and high Richardson numbers. Alinia et al.^28^ examined the mixed convection of a nanofluid having water and $\text {Si}\text {O}_2$ into a tilted enclosure. The authors concluded that nanoparticles’ inclusion significantly improved heat transfer in the cavity and induced significant alterations within the flow distribution. Shariat et al.^29^ investigated the two-phase mixture model on laminar mixed convection alumina-water nanofluid flow inside elliptic ducts with a fixed heat flux boundary. The results indicated that increasing the nanoparticle volume fraction improved the Nusselt number for a given Reynolds and Richardson number while decreasing the skin friction.

In addition to studies using nanofluids and various types of cavities, studies on heat transfer and fluid flow inside cavities with an inner body have experienced significant awareness during recent years due to their practical engineering purposes. Free convection in cavities having a square inner solid was previously discussed in studies by Alsabery et al.^30^, Alsabery et al.^31^, Mahmoodi and Sebdani^32^, and Sheremet et al.^33^. The influence of nanoparticles’ addition on the heat transfer rate was necessary for the low Rayleigh number and number of undulations^34^. Alsabery et al.^35^ investigated the time-dependent mixed convection of alumina-water nanoliquid inside the differentially-heated chamber having a wavy upper surface and central solid cylinder. The findings discovered that the average Nusselt number of the hot surface improved on the moving parameter and inner solid cylinder diameter. Liao and Lin^36^ investigated mixed convection within the square cavity which contains an isothermally-rotating cylinder. It was observed that the rate of heat transfer depends on the Rayleigh number, aspect ratio, and Prandtl number. The study on the MHD-mixed convection of nanofluids (Cu–water) inside a triangular cavity including a rotating cylinder was conducted by Shariat et al.^37^. It was observed that heat transfer and total entropy production increased as the nanoparticles’ solid volume fraction improved. Billah et al.^38^ investigated the mixed convection into a lid-driven cavity containing a heated cylinder placed at the centre of the cavity. The results indicated that the flow field and temperature distribution were remarkably dependent on the used cylinder’s diameter. Recently, Alhashash^39^ employed the nonhomogeneous two-phase Buongiorno’s model to study the effect of an inner cylinder on natural convection in a square porous cavity with all walls kept cold. Chatterjee et al.^40^ were probably the first to examine the simultaneous effects of a moving lid and an inner rotating cylinder on the mixed convective transport of nanofluids ($\text {Cu-H}_2\text {O}$) in a bottom-heated square enclosure. Alsabery et al.^41^ considered the effect of an inner rotating cylinder on entropy generation and convective heat transfer in a wavy porous cavity partially-heated from below. The entropy production and mixed convection inside a wavy-walled, nanofluid-filled cavity which is partially-heated from below and contains an inner rotating conductive cylinder was investigated by Alsabery et al.^42^. The authors showed that the cylinder rotation increased the rate of heat exchange for a certain Rayleigh number and increased with the nanoparticle volume fraction of the heater segment’s length.

Based on the studies mentioned above and to the best of our knowledge, no investigation has been performed on entropy generation and mixed convection of nanofluids within a lid-driven trapezoidal cavity containing an inner solid cylinder. The heat and fluid flow can be affected significantly by the inclusion of an inner body (like a square or a cylinder) without consuming extra energy. Therefore, this investigation intends to examine the influence of solid cylinder’s location on the entropy production and mixed convection inside a lid-driven trapezoidal cavity having water–$\hbox {Al}_2\hbox {O}_3$ nanofluids. The outcomes shall be reported for various Reynolds number, Richardson number, nanoparticles volume fraction, the solid cylinder’s dimensionless radius and location toward streamlines, isotherms, isentropic, local and average Nusselt numbers, Bejan number, and the global entropy generation.

## Mathematical formulation

**Figure 1:**
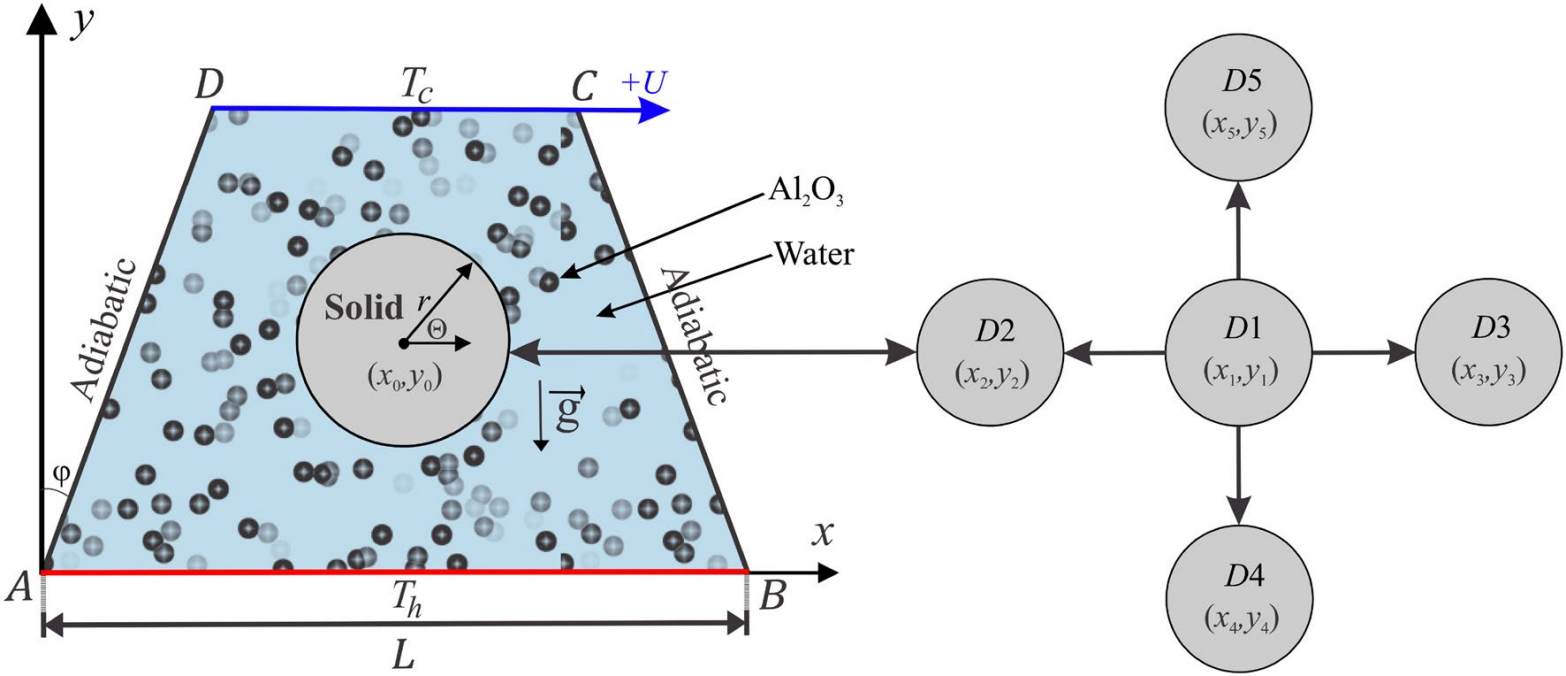
Physical model of convection in a trapezoidal cavity together with the conducting wall and coordinate systems.

The two-dimensional geometric design regarding mixed convection flow and heat transfer inside a trapezoidal cavity with a bottom wall of length *L* and top wall with range *L*/2, containing an internal solid cylinder with dimensional radius *r* that is in various locations (D1–D5) is described schematically in Figure 1. The bottom surface is assumed to be heated with a constant temperature of $T_{h}$, and the top surface slide has uniform velocity from left to right at $+U$ and is preserved at a constant cold temperature, $T_{c}$. Both sloping surfaces with length 0.65*L* and inclination angle $\varphi$ are maintained under adiabatic processes. The fluid flow in the trapezoidal cavity was examined to hold steady, laminar, and loaded with alumina–water nanofluids. The governing equations of Navier–Stokes and energy equations toward the viscous incompressible flow are indicated in the dimensional model as the following^43^:

1 $$\begin{aligned}&\frac{{\partial u}}{{\partial x}} + \frac{{\partial v}}{{\partial y}} = 0, \end{aligned}$$

2 $$\begin{aligned}&u\frac{{\partial u}}{{\partial x}} + v\frac{{\partial u}}{{\partial y}} = - \frac{1}{{{\rho _{nf}}}}\frac{{\partial p}}{{\partial x}} + {\nu _{nf}}\,\left( {\frac{{{\partial ^2}u}}{{\partial {x^2}}} + \frac{{{\partial ^2}u}}{{\partial {y^2}}}} \right) , \end{aligned}$$

3 $$\begin{aligned}&u\frac{{\partial v}}{{\partial x}} + v\frac{{\partial v}}{{\partial y}} = - \frac{1}{{{\rho _{nf}}}}\frac{{\partial p}}{{\partial y}} + {\nu _{nf}}\,\left( {\frac{{{\partial ^2}v}}{{\partial {x^2}}} + \frac{{{\partial ^2}v}}{{\partial {y^2}}}} \right) + \beta _{nf} \, g(T - {T_0}), \end{aligned}$$

4 $$\begin{aligned}&u\frac{{\partial T}}{{\partial x}} + v\frac{{\partial T}}{{\partial y}} = {\alpha _{nf}}\left( {\frac{{{\partial ^2}T}}{{\partial {x^2}}} + \frac{{{\partial ^2}T}}{{\partial {y^2}}}} \right) . \end{aligned}$$

The heat equation of the solid inner body remains as:

5 $$\begin{aligned} \frac{{{\partial ^2}T_s}}{{\partial {x^2}}} + \frac{{{\partial ^2}T_s}}{{\partial {y^2}}} = 0, \end{aligned}$$

where *x* and *y* are the Cartesian coordinates measured in the horizontal and vertical directions respectively, *g* is the acceleration due to gravity, $\rho _{nf}$ is the density of the nanofluid and $\nu _{nf}$ is the kinematic viscosity of the nanofluid.

The following relations can describe the thermophysical features of the applied nanofluids^44^:

6 $$\begin{aligned}&(\rho C_p)_{nf}=(1-\phi )(\rho C_p)_{f}+\phi (\rho C_p)_{p}, \end{aligned}$$

7 $$\begin{aligned}&\alpha _{nf}=\frac{k_{nf}}{(\rho C_p)_{nf}}, \end{aligned}$$

8 $$\begin{aligned}&\rho _{nf}=(1-\phi )\rho _{f}+\phi \rho _{p}, \end{aligned}$$

9 $$\begin{aligned}&{(\rho \beta )_{nf}} = (1 - \phi ){(\rho \beta )_{f}} + \phi {(\rho \beta )_{p}}, \end{aligned}$$

10 $$\begin{aligned}&\frac{\mu _{nf}}{\mu _f} = \frac{1}{1-34.87\left( \frac{d_p}{d_f}\right) ^{-0.3}\phi ^{1.03}}, \end{aligned}$$

11 $$\begin{aligned}&\frac{k_{nf}}{k_{f}}= 1 + 4.4 \text {Re}_B^{0.4} \text {Pr}^{0.66} \left( \frac{T}{T_{fr}}\right) ^{10} \left( \frac{k_p}{k_f}\right) ^{0.03} \phi ^{0.66}. \end{aligned}$$

Here $\text {Re}_B$ is defined as

12 $$\begin{aligned} \text {Re}_B=\frac{\rho _f u_B d_p}{\mu _f}, \quad u_B = \frac{2k_b T}{\pi \mu _f d_p^2}, \end{aligned}$$

while the molecular diameter of water ($d_f$) is defined by the following equation^44^:

13 $$\begin{aligned} d_f = 0.1 \left[ \frac{6 M}{N \pi \rho _f}\right] ^{\frac{1}{3}}. \end{aligned}$$

The following non-dimensional variables are introduced as follows:

14 $$\begin{aligned}&X=\frac{x}{L},\quad Y=\frac{y}{L},\quad U = \frac{u}{U_{0}}, \,\, V = \frac{v}{U_{0}},\quad \theta =\frac{T - T_c}{T_h - T_c},\quad \theta _s=\frac{T_s - T_c}{T_h - T_c},\nonumber \\&\quad D=\frac{d}{L}, \quad \Pr = \frac{\nu _f}{\alpha _f}, \quad P=\frac{pL^2}{\rho _{f} \alpha _f^2}. \end{aligned}$$

This then yields the dimensionless governing equations as follows:

15 $$\begin{aligned}&\frac{{\partial U}}{{\partial X}} + \frac{{\partial V}}{{\partial Y}} = 0\! \end{aligned},$$

16 $$\begin{aligned}&U\frac{{\partial U}}{{\partial X}} + V\frac{{\partial U}}{{\partial Y}} = - \frac{{\partial P}}{{\partial X}} + \frac{1}{Re} \frac{\rho _{f}}{\rho _{nf}} \frac{\mu _{nf}}{\mu _{f}} \left( {\frac{{{\partial ^2}U}}{{\partial {x^2}}} + \frac{{{\partial ^2}U}}{{\partial {Y^2}}}} \right) \end{aligned}\!,$$

17 $$\begin{aligned}&U\frac{{\partial V}}{{\partial X}} + V\frac{{\partial V}}{{\partial Y}} = - \frac{{\partial P}}{{\partial Y}} + \frac{1}{Re} \frac{\rho _{f}}{\rho _{nf}} \frac{\mu _{nf}}{\mu _{f}} \left( {\frac{{{\partial ^2}V}}{{\partial {X^2}}} + \frac{{{\partial ^2}V}}{{\partial {Y^2}}}} \right) + \frac{(\rho \beta )_{nf}}{\rho _{nf} \beta _{f}} \frac{Gr}{Re^{2}} {\theta } \end{aligned}\!,$$

18 $$\begin{aligned}&U\frac{{\partial \theta }}{{\partial X}} + V\frac{{\partial \theta }}{{\partial Y}} = \frac{\alpha _{nf}}{\alpha _{f}} \frac{1}{{\Pr }\, {Re}} \left( \frac{{{\partial ^2}\theta }}{{\partial {X^2}}} + \frac{{{\partial ^2}\theta }}{{\partial {Y^2}}}\right) , \end{aligned}$$

19 $$\begin{aligned}&\frac{{{\partial ^2}\theta _s}}{{\partial {x^2}}} + \frac{{{\partial ^2}\theta _s}}{{\partial {y^2}}} = 0, \end{aligned}$$

where $Ri=\frac{Gr}{Re^{2}}$ is the Richardson number and $Gr=\frac{g \beta _f \left( T_h-T_c \right) L^3}{\nu _{f}^2}$ is the Grashof number. The dimensionless boundary conditions as indicated in Eqs. (15) and (19) are shown below:

20 $$\begin{aligned}&\text {On the bottom heated surface} (AB): \nonumber \\&U = V = 0,\,\, \theta =1, \,\, 0\le X\le 1,\,\, Y=0, \end{aligned}$$

21 $$\begin{aligned}&\text {On the top moving cold surface} (DC): \nonumber \\&U = 1, \, V = 0,\,\, \theta =0,\,\, 0\le X \le 1,\,\, Y=0.65, \end{aligned}$$

22 $$\begin{aligned}&\text {On the left and right sloping surfaces} (AD \, \text {and} \, BC): \nonumber \\&U = V = 0,\,\, \frac{\partial \theta }{\partial (X,Y)}=0,\,\, \forall {X},\,\, \forall {Y}, \end{aligned}$$

23 $$\begin{aligned}&\theta = {\theta _{s}},\,\, \text {at the outer solid cylinder surface}, \end{aligned}$$

24 $$\begin{aligned}&U = V = 0,\,\, \frac{\partial \theta }{\partial n} = K_{r}\frac{\partial \theta _{s}}{\partial n}, \end{aligned}$$

where $K_{r} = {k_s}/{k_{nf}}$ keeps the thermal conductivity ratio above the covering of the internal body. The local Nusselt number estimated through the heated base horizontal surface does represent by:

25 $$\begin{aligned} Nu_{nf} = - \left( \frac{\partial \theta }{\partial X}\right) _{Y=0}. \end{aligned}$$

Finally, the average Nusselt number could be determined through the heated segment of the bottom horizontal surface of the cavity, which does represent by:

26 $$\begin{aligned} {\overline{Nu}}_{nf} = \int _{A}^{B} Nu_{nf} {} \mathrm{{d}}Y. \end{aligned}$$

The entropy generation relation as indicated by^41,45^ is shown in dimensionless form below:

27 $$\begin{aligned} S_{\text {GEN}}& {} = \frac{k_{nf}}{k_f} \left[ \left( \frac{\partial \theta }{\partial X}\right) ^2 + \left( \frac{\partial \theta }{\partial Y}\right) ^2 \right] \nonumber \\&\quad + \frac{\mu _{nf}}{\mu _f} N_\mu \left\{ 2 \left[ \left( \frac{\partial U}{\partial X}\right) ^2 + \left( \frac{\partial V}{\partial Y}\right) ^2 \right] + \left( \frac{\partial ^2 U}{\partial Y^2} + \frac{\partial ^2 V}{\partial X^2} \right) ^2 \right\} , \end{aligned}$$

where $N_\mu =\frac{\mu _f T_0}{k_f} \left( \frac{\alpha _f}{L(\Delta T)}\right) ^2$ is the irreversibility distribution ratio and $S_{\text {GEN}}=S_{gen}\frac{T_0^2 L^2}{k_f (\Delta T)^2}$. The terms of Eq. (27) can be separated based on the following formula:

28 $$\begin{aligned} S_{\text {GEN}}= S_\theta + S_\Psi , \end{aligned}$$

where $S_\theta$ and $S_\Psi$ are the entropy generation due to heat transfer irreversibility (HTI) and fluid friction irreversibility (FFI), respectively,

29 $$\begin{aligned}&S_\theta = \frac{k_{nf}}{k_f} \left[ \left( \frac{\partial \theta }{\partial X}\right) ^2 + \left( \frac{\partial \theta }{\partial Y}\right) ^2 \right] , \end{aligned}$$

30 $$\begin{aligned}&S_\Psi = \frac{\mu _{nf}}{\mu _f} N_\mu \left\{ 2 \left[ \left( \frac{\partial U}{\partial X}\right) ^2 + \left( \frac{\partial V}{\partial Y}\right) ^2 \right] + \left( \frac{\partial ^2 U}{\partial Y^2} + \frac{\partial ^2 V}{\partial X^2} \right) ^2 \right\} . \end{aligned}$$

By integrating Eq. (28) over the domain, the global entropy generation (GEG) for the present two-dimensional study is obtained as follows:

31 $$\begin{aligned} \text {GEG} = \int S_{\text {GEN}} {} \mathrm{{d}}X \mathrm{{d}}Y = \int S_\theta {} \mathrm{{d}}X \mathrm{{d}}Y + \int S_\Psi {} \mathrm{{d}}X \mathrm{{d}}Y. \end{aligned}$$

It is necessary to mention the Bejan number in determining which is dominant, either heat transfer or fluid friction irreversibility. Bejan number is defined as:

32 $$\begin{aligned} Be = \frac{\int S_\theta \mathrm{{d}}X \mathrm{{d}}Y}{\int S_{\text {GEN}} \mathrm{{d}}X \mathrm{{d}}Y}. \end{aligned}$$

The HTI is dominant when $Be > 0.5$, while the FFI is dominant when $Be < 0.5$.

## Numerical method and validation

The Galerkin weighted residual with finite element methods was employed to investigate the control equations (15)–(19) subject to the boundary conditions shown in Eqs. (20)–(24). The finite element analysis of the momentum equations (16) and (17) is represented by the following procedure:

Firstly, the penalty finite element method was applied by excluding the pressure (*P*) and including a penalty parameter ($\lambda$) as follows:

$$\begin{aligned} P=-\lambda \left( \frac{\partial U}{\partial X}+\frac{\partial V}{\partial Y}\right) . \end{aligned}$$

The following momentum equations toward the *X* and *Y*-directions ares shown as follows:

$$\begin{aligned}&U\frac{\partial U}{\partial X} + V\frac{\partial U}{\partial Y} = \frac{\partial \lambda }{\partial X} \left( \frac{\partial U}{\partial X}+\frac{\partial V}{\partial Y}\right) + \frac{\rho _{f}}{\rho _{nf}} \frac{\mu _{nf}}{\mu _{f}} \frac{1}{Re} \left( {\frac{\partial ^2 U}{\partial X^2} + \frac{\partial ^2 U}{\partial Y^2}}\right) , \nonumber \\&U\frac{\partial V}{\partial X} + V\frac{\partial V}{\partial Y} = \frac{\partial \lambda }{\partial Y} \left( \frac{\partial U}{\partial X}+\frac{\partial V}{\partial Y}\right) + \frac{\rho _{f}}{\rho _{nf}} \frac{\mu _{nf}}{\mu _{f}} \frac{1}{Re} \left( {\frac{\partial ^2 V}{\partial X^2} + \frac{\partial ^2 V}{\partial Y^2}}\right) + \frac{(\rho \beta )_{nf}}{\rho _{nf} \beta _{f}} \frac{Gr}{Re^{2}} {\theta _{nf}}. \end{aligned}$$

**Figure 2:**
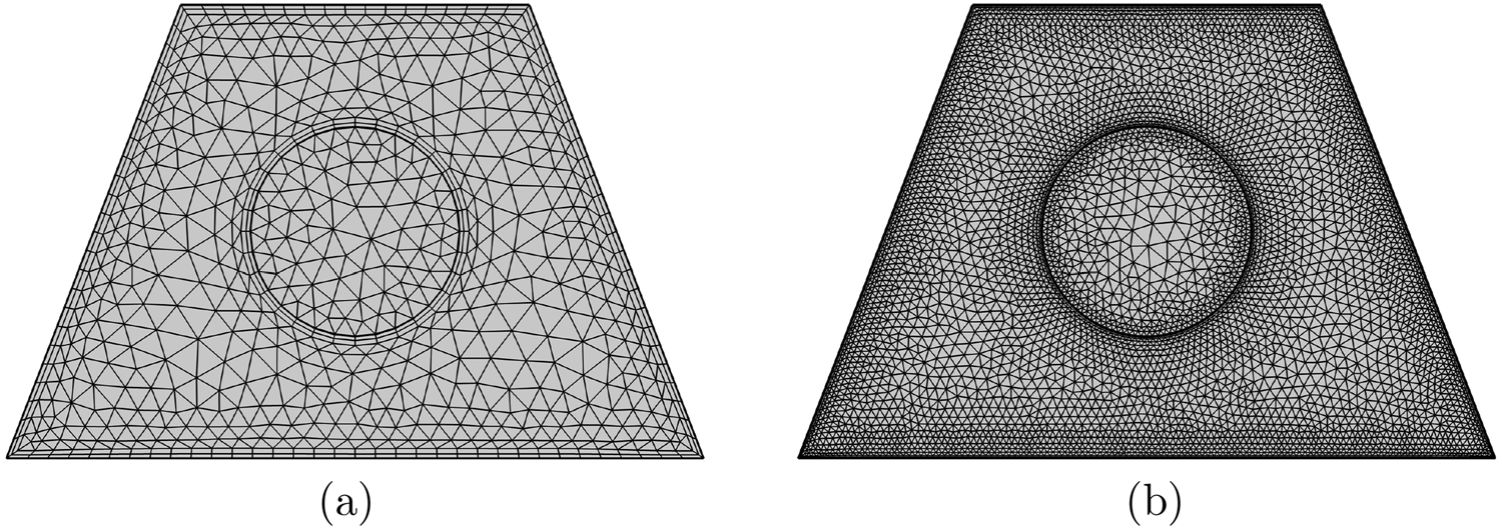
Grid-points distribution for the grid size of (**a**) 1339 and (**b**) 9846 elements.

The weak (or weighted-integral) formulation regarding the momentum equations was obtained by multiplying the equation by an internal domain ($\Phi$) and integrating it over the computational domain which is discretised towards small triangular elements as revealed in Fig. 2. The following weak formulations are obtained:

$$\begin{aligned}&\int _{\Omega } \left( \Phi _i U^k \frac{\partial U^k}{\partial X} + \Phi _i V^k \frac{\partial U^k}{\partial Y}\right) \mathrm{{d}X\mathrm {d}Y}= \lambda \int _{\Omega } \frac{\partial \Phi _i}{\partial X} \left( \frac{\partial U^k}{\partial X}+\frac{\partial V^k}{\partial Y}\right) \mathrm{{d}X\mathrm {d}Y} \\&\quad + \frac{\rho _{f}}{\rho _{nf}} \frac{\mu _{nf}}{\mu _{f}} \frac{1}{Re} \int _{\Omega }\Phi _i \left( \frac{\partial ^2 U^k}{\partial X^2} + \frac{\partial ^2 U^k}{\partial Y^2}\right) \mathrm{{d}X\mathrm {d}Y}, \\&\int _{\Omega } \left( \Phi _i V^k \frac{\partial V^k}{\partial X} + \Phi _i V^k \frac{\partial V^k}{\partial Y}\right) \mathrm{{d}X\mathrm {d}Y}= \lambda \int _{\Omega } \frac{\partial \Phi _i}{\partial Y} \left( \frac{\partial U^k}{\partial X}+\frac{\partial V^k}{\partial Y}\right) \mathrm{{d}X\mathrm {d}Y} \\&\quad \quad + \frac{\rho _{f}}{\rho _{nf}} \frac{\mu _{nf}}{\mu _{f}} \frac{1}{Re} \int _{\Omega }\Phi _i \left( \frac{\partial ^2 V^k}{\partial X^2} + \frac{\partial ^2 V^k}{\partial Y^2}\right) \mathrm{{d}X\mathrm {d}Y} + \frac{(\rho \beta )_{nf}}{\rho _{nf} \beta _{f}} \frac{Gr}{Re^{2}} \int _{\Omega }\Phi _i \theta _{nf}^k\mathrm{{d}X\mathrm {d}Y}. \end{aligned}$$

The selection of interpolation functions as an approximation towards velocity distribution and temperature distribution is as follows:

$$\begin{aligned}&U \approx \sum _{j=1}^m U_j \Phi _j(X,Y), \quad V \approx \sum _{j=1}^m V_j \Phi _j(X,Y), \quad \theta \approx \sum _{j=1}^m \theta _j \Phi _j(X,Y). \end{aligned}$$

The non-linear residual equations for the momentum equations obtained from the Galerkin weighted residual finite-element method are as follows:

$$\begin{aligned} R(1)_i& {} = \sum _{j=1}^{m}U_j \int _{\Omega } \left[ \left( \sum _{j=1}^{m}U_j\Phi _j\right) \frac{\partial \Phi _j}{\partial X} + \left( \sum _{j=1}^{m}V_j\Phi _j\right) \frac{\partial \Phi _j}{\partial Y} \right] \Phi _i\mathrm{{d}X\mathrm {d}Y}\\&\quad + \lambda \left[ \sum _{j=1}^{m} U_j \int _{\Omega }\frac{\partial \Phi _i}{\partial X} \frac{\partial \Phi _j}{\partial X}\mathrm{{d}X\mathrm {d}Y} + \sum _{j=1}^{m} V_j \int _{\Omega }\frac{\partial \Phi _i}{\partial X} \frac{\partial \Phi _j}{\partial Y}\mathrm{{d}X\mathrm {d}Y}\right] \\&\quad + \frac{\rho _{f}}{\rho _{nf}} \frac{\mu _{nf}}{\mu _{f}} \frac{1}{Re} \sum _{j=1}^{m}U_j \int _{\Omega } \left[ \frac{\partial \Phi _i}{\partial X}\frac{\partial \Phi _j}{\partial X} + \frac{\partial \Phi _i}{\partial Y}\frac{\partial \Phi _j}{\partial Y}\right] \mathrm{{d}X\mathrm {d}Y}, \\ R(2)_i& {} = \sum _{j=1}^{m}V_j \int _{\Omega } \left[ \left( \sum _{j=1}^{m}U_j\Phi _j\right) \frac{\partial \Phi _j}{\partial X} + \left( \sum _{j=1}^{m}V_j\Phi _j\right) \frac{\partial \Phi _j}{\partial Y} \right] \Phi _i\mathrm{{d}X\mathrm {d}Y}\\&\quad + \lambda \left[ \sum _{j=1}^{m} U_j \int _{\Omega }\frac{\partial \Phi _i}{\partial Y} \frac{\partial \Phi _j}{\partial X}\mathrm{{d}X\mathrm {d}Y} + \sum _{j=1}^{m} V_j \int _{\Omega }\frac{\partial \Phi _i}{\partial Y} \frac{\partial \Phi _j}{\partial Y}\mathrm{{d}X\mathrm {d}Y}\right] \\&\quad + \frac{\rho _{f}}{\rho _{nf}} \frac{\mu _{nf}}{\mu _{f}} \frac{1}{Re} \sum _{j=1}^{m}V_j \int _{\Omega } \left[ \frac{\partial \Phi _i}{\partial X}\frac{\partial \Phi _j}{\partial X} + \frac{\partial \Phi _i}{\partial Y}\frac{\partial \Phi _j}{\partial Y}\right] \mathrm{{d}X\mathrm {d}Y} \\&\quad + \frac{(\rho \beta )_{nf}}{\rho _{nf} \beta _{f}} \frac{Gr}{Re^{2}} \int _{\Omega } \left( \sum _{j=1}^{m}\theta _j \Phi _j\right) \Phi _i\mathrm{{d}X\mathrm {d}Y}, \end{aligned}$$

where the superscript *k* is the relative index, subscripts *i*, *j*, and *m* are the residual number, node number, and iteration number, respectively. For clarifying the non-linear terms into the momentum equations, a Newton-Raphson iteration algorithm was employed. The convergence of the solution was allowed using the relative error to any of the variables that satisfy the resulting convergence criteria as follows:

$$\begin{aligned} \left| \frac{\Gamma ^{m+1}-\Gamma ^{m}}{\Gamma ^{m+1}}\right| \le 10^{-5}. \end{aligned}$$

To assure the confidence of the existing numerical solution at the grid dimension of the adopted domain, different grid sizes have been applied to calculate the average Nusselt number and Bejan number concerning the case of $Ri=1$, $Re=100$, $\phi =0.02$, $R=0.15$ and *D*1. The outcomes are exhibited in Table 1 which show insignificant variations for the G5 grids and above. Hence, for all calculations in the current problem, the G5 uniform grid remains used.

**Table 1:** Grid testing for ${\overline{Nu}}_{nf}$ and *Be* at different grid sizes for $Ri=1$, $Re=100$, $\phi =0.02$, $R=0.15$ and *D*1.

Grid size	Number of elements	${\overline{Nu}}_{nf}$	*Be*
G1	757	5.7418	0.98632
G2	1339	6.0999	0.9828
G3	2078	6.261	0.98061
G4	3077	6.3703	0.97899
**G5**	9846	6.5638	0.97567
G6	26969	6.5688	0.97417

**Figure 3:**
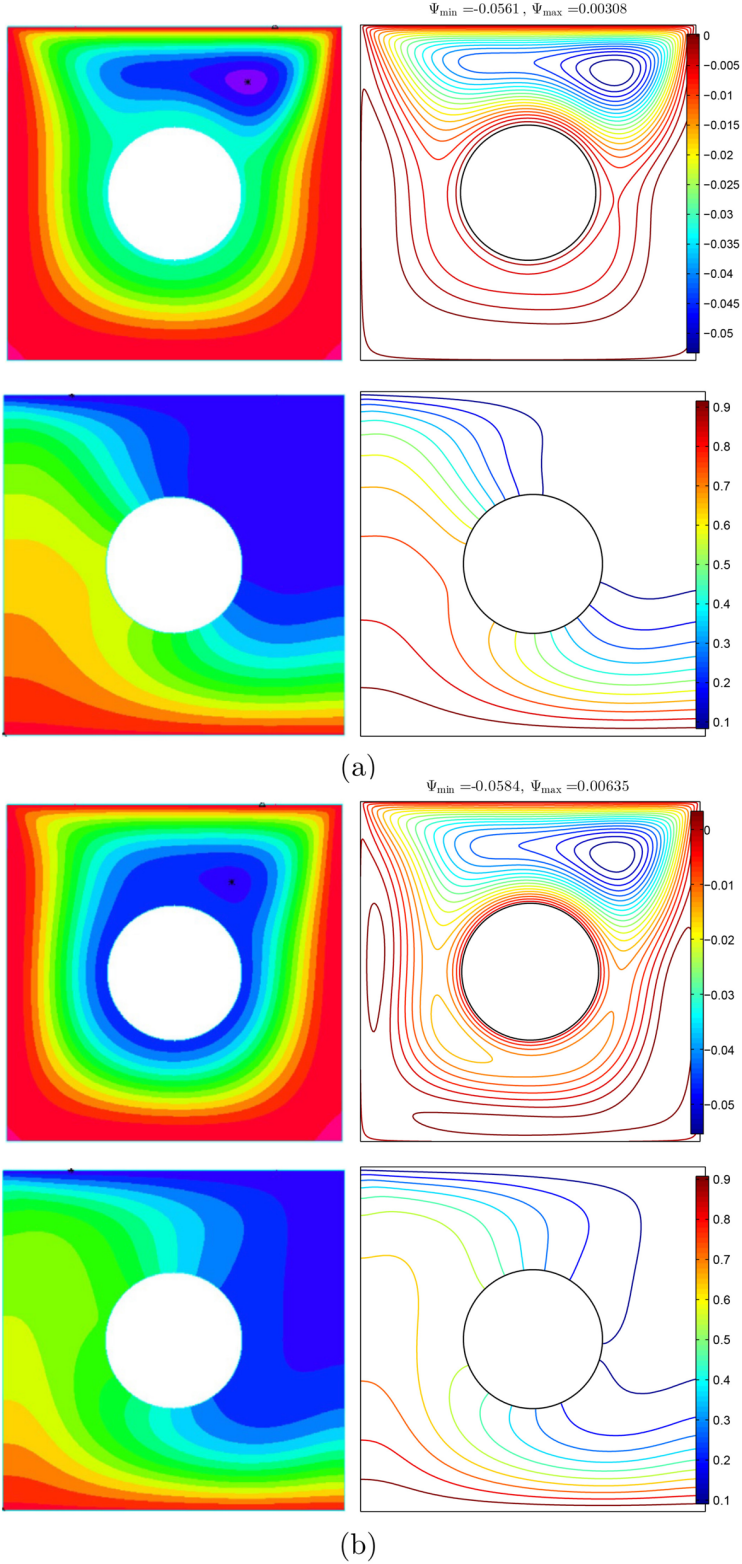
Comparisons of results by Khanafer and Aithal^43^ (left) and present study (right) for (**a**) streamlines at $Ra=10^{6}$ and $H=0.4$, (**b**) isotherms at $Ra=10^{5}$ and $H=0.8$, and (**c**) isotherms at $Ra=1.836\times 10^{5}$ and $H=0.8$ for numerical and experimental results obtained by Khanafer and Aithal^43^ at $\phi =0$ and $D=0$.

**Figure 4:**
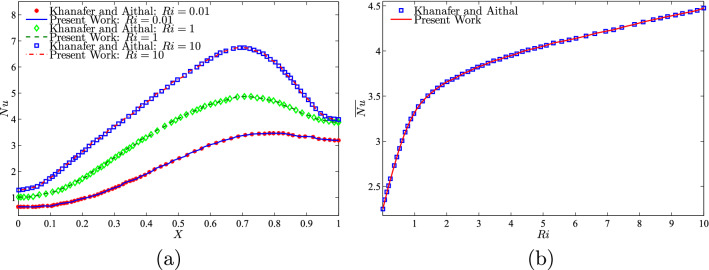
Comparison of the average Nusselt number interface with *Ra* for different *H* values with results by Khanafer and Aithal^43^ at $\phi =0$ and $D=0$.

**Figure 5:**
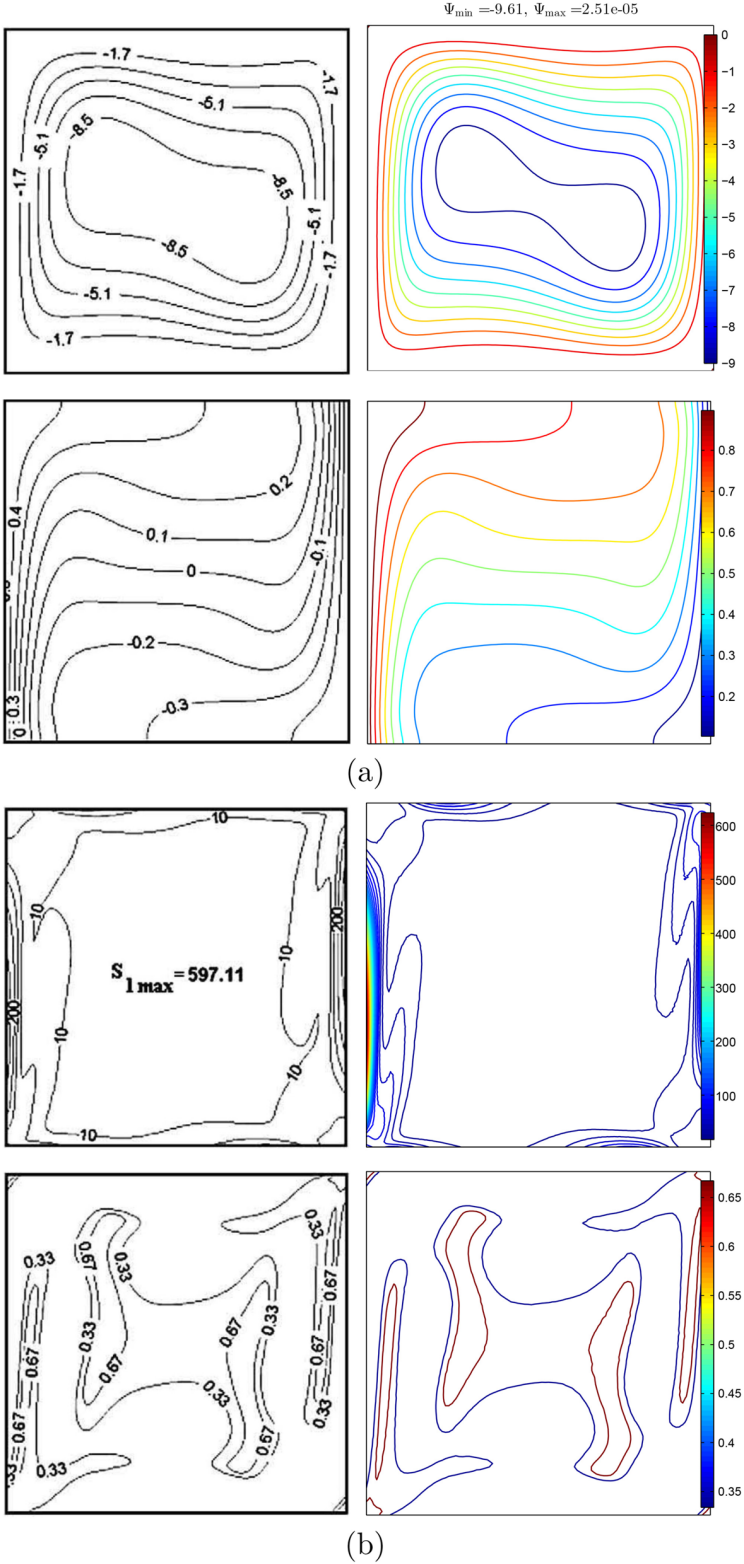
Comparisons of streamlines and isotherms (**a**), and global entropy generation and Bejan number (**b**) for $Ra=10^{5}$, $\phi =0$, *D* and $H=1$ based on results obtained by Ilis et al.^45^ (left) and this study (right).

**Figure 6:**
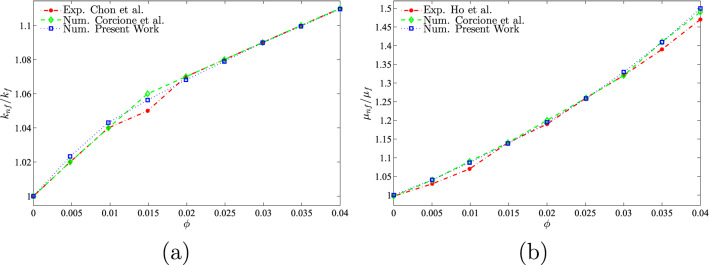
Comparison of (**a**) thermal conductivity ratio with results obtained by Chon et al.^47^ and Corcione et al.^46^ and (**b**) dynamic viscosity ratio with results obtained by Ho et al.^48^ and Corcione et al.^46^.

For data validation, the results obtained in this study were compared with previous numerical findings presented by Khanafer and Aithal^43^ for free convective flow and heat transfer in a square cavity filled with pure fluid and partial heating from below as shown, in Figs. 3 and 4. In addition, a comparison was made between the resulting figures and the one provided by Ilis et al.^45^ for entropy generation and natural convection in a square cavity fully heated from the side walls as shown in Fig. 5. Figure 6 shows alternative comparisons for the enhancement in the thermal conductivity due to the addition of $\text {Al}_2\text {O}_3$ nanoparticles with two different experimental results and the numerical results obtained by Corcione et al.^46^. These results provide support for the accuracy obtained using the numerical method illustrated in this study.

## Results and discussion

The numerical results for the streamlines, isotherms, and isentropic lines (the local dimensionless entropy generation) along with various values for Richardson number $(0.01\le Ri\le 10)$, Reynolds number ($5\le Re \le 500$), nanoparticles volume fraction ($0\le \phi \le 0.04$), dimensionless radius of solid cylinder ($0.05 \le S \le 0.25$) and dimensionless location of solid cylinder (*D*) [$D1=(X=0.5,Y=0.5),D2=(X=0.35,Y=0.5),D3=(X=0.65,Y=0.5),D4=(X=0.5,Y=0.2),D5=(X=0.5,Y=0.45)$] are presented in this section. The values for Prandtl number, thermal conductivity of the solid cylinder and side surface inclination angle are fixed at $\Pr =4.623$, $k_{s}=0.76$ and $\varphi =22.5^\circ$ respectively. The thermophysical properties of the used base fluid (water) and solid $\hbox {Al}_2$ $\hbox {O}_3$ phases are shown in Table 2.

**Table 2:** Thermo-physical properties of water with $\hbox {Al}_{{2}}$ $\hbox {O}_{{3}}$ nanoparticles at $T=310$K^49^.

Physical properties	Fluid phase (water)	$\hbox {Al}_{{2}}$ $\hbox {O}_{{3}}$
$C_p\, \text {(J/kg K)}$	4178	765
$\rho \, (\text {kg/m}^3)$	993	3970
$k\, (\text {W m}^{-1}\,\text {K}^{-1})$	0.628	40
$\beta \times 10^{-5}\, \text {(1/K)}$	36.2	0.85
$\mu \times 10^{-6}\, \text {(kg/ms)}$	695	–
$d_p\, \text {(nm)}$	0.385	33

**Figure 7:**
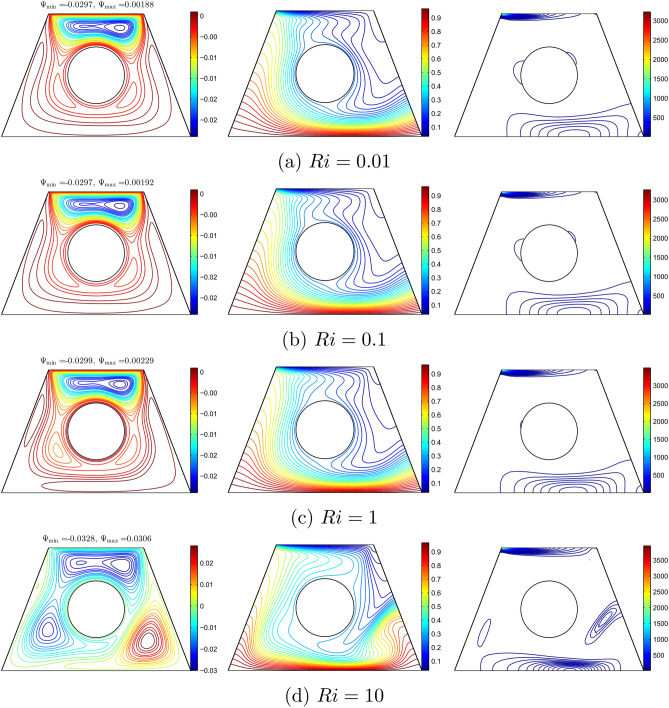
Variation of the streamlines (left), isotherms (middle), and isentropic (right) evolution based on the Richardson number (*Ri*) for $Re=100$, $\phi =0.02$, $R=0.15$ and *D*1.

Figure 7 shows the distributions of streamlines, isotherms, and isentropic lines for different values of Richardson number for $\phi =0.02$, $R=0.15$, $Re=100$, and the inner solid located at the centre of the solid. Therefore, the increase in *Ri* is due to the increase in the Grashof number. Natural convection is negligible if the Richardson number is less than 1.0. Due to the motion direction of the upper wall, one small cell appeared at the top of the solid wall in Fig. 7(a,b). As *Ri* increase, the streamlines extend downwards as shown in Fig. 7(c) since both natural and forced convection exist. When *Ri* increases to 10, multi-cellular patterns appear at the top of the wall as well as the bottom of the wall. Since the bottom wall is heated and the upper wall is cooled, the isotherm patterns appear as curved lines. It can be observed that the curved lines appear at the top left and the bottom right of the solid as in Fig. 7(a). No change in the isotherm patterns was observed as *Ri* increased. However it was observed that the isotherm curved lines elongated to the right of the solid cylinder at $Ri=10$. The entropy regions are produced by the heat transfer irreversibility (HTI) and the nanofluid flow irreversibility (NFI) that occur at high temperatures and the velocity gradients. The isentropic lines depict a concentrated entropy generation close to three regions, namely at the top and right bottom of the enclosure and the surface of the solid cylinder.No change was observed for the isentropic lines at a smaller *Ri* number. However, the isentropic lines changed slightly at $Ri=10$ as the lines appeared on the bottom (centre) of the solid and the sloping surfaces of the wall.

**Figure 8:**
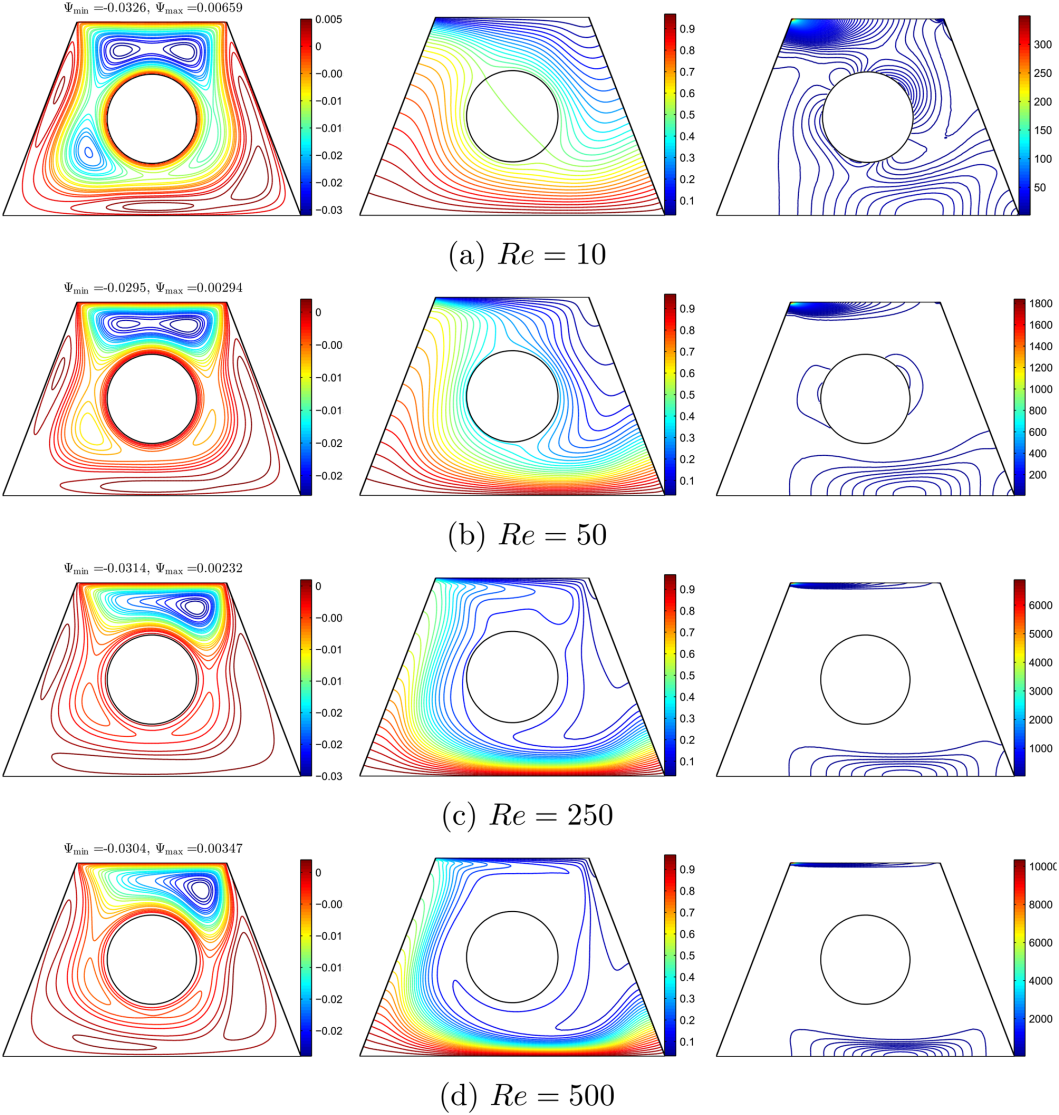
Variation of the streamlines (left), isotherms (middle), and isentropic (right) evolution based on the Reynolds number (*Re*) for $Ri=1$, $\phi =0.02$, $R=0.15$ and *D*1.

Figure 8 shows the effect of various Reynold numbers on streamlines, isotherm, and isentropic with $Ri=1$, $\phi =0.02$, and $R=0.15$. In Figure 8(a), the streamlines appear with two rotating cells at the top of the wall and one rotating cell at the bottom of the wall. This is due to the shear force and buoyant force that dominate at a smaller Reynolds number.As *Re* increases, the cell at the bottom of the wall and the top right shrink, while the cell at the top left expands as shown in Figure 8(b). Furthermore, the cells at the top right and bottom do not exist, while the cell at the top left expands at higher *Re* as shown in Figure 8(c) and (d). This is because the velocity of the top lid increases as the Reynolds number increases. Since the velocity of the top lid has increased, forced convection is more dominant as compared to the buoyant force. Since the cooling activity occurs at the top wall and the heating activity occurs at the bottom wall, the thick thermal boundary layers are clustered at the top left and bottom right of the cavity wall. The isotherms are approximately horizontal. The increase in *Re* disrupts the isotherm inside the cavity. At $Re=50$, it can be observed that the intensity of the isotherm patterns increased at the top left and bottom right walls and decreased at the right side of the cavity. Furthermore, the intensity of the isotherm pattern decreased in the cavity as *Re* increases. The isentropic lines indicate a concentrated entropy generation inside the cavity and around the solid cylinder. These entropy regions result from heat transfer irreversibility (HTI) and nanofluid irreversibility (NFI) that arise from shear force and buoyant force. As *Re* increases, the intensity of isentropic lines decreases, especially around the solid cylinder. At $Re=500$, the isentropic lines only appear at the top and bottom of the wall as the intensity of isentropic lines was high at the top of the wall due to the dominant shear force as *Re* increased.

**Figure 9:**
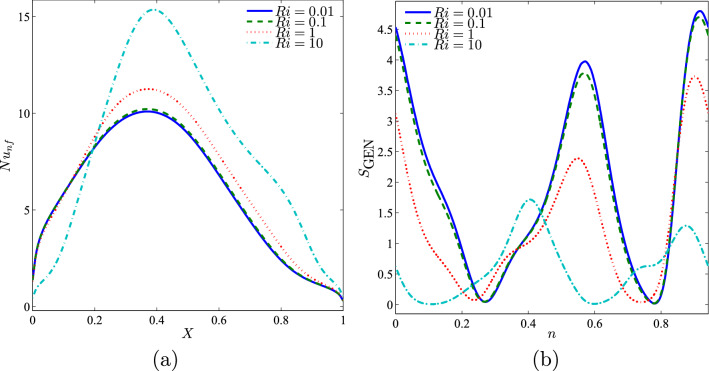
Variation of (**a**) local Nusselt number interfaces with *X* and (**b**) local entropy generation at the outer solid cylinder surface for different *Ri* values at $Re=100$, $\phi =0.02$, $R=0.15$, and *D*1.

Figure 9 depicts the local Nusselt number interface with *X* and the local entropy generation at the outer cylinder surface for different Richardson numbers. The local Nusselt number was maximum for all Richardson number at $X=0.4$. The highest local Nusselt number was obtained when $Ri=10$ as the system became dominant with natural convection. At the lowest Richardson number, the local entropy generation was highest at both the vertical walls.

**Figure 10:**
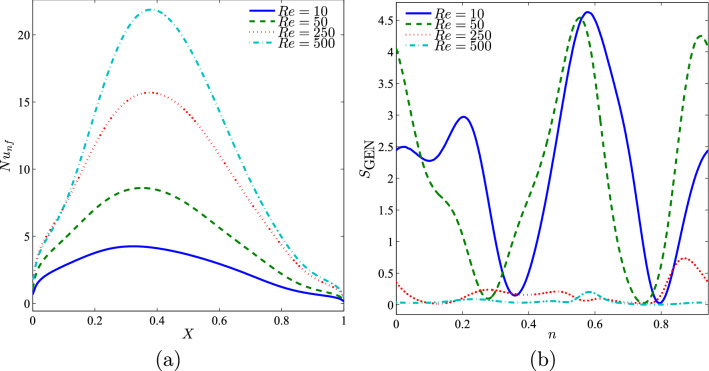
Variation of (**a**) local Nusselt number interfaces with *X* and (**b**) local entropy generation at the outer solid cylinder surface for different *Re* values at $Ri=1$, $\phi =0.02$, $R=0.15$, and *D*1.

Figure 10 depicts the distribution of the local Nusselt number, in which the maximum heat transfer was exhibited at $X=0.4$ for all the Reynolds number. A higher Reynolds number provides maximum heat transfer as compared to a smaller Reynolds number.

**Figure 11:**
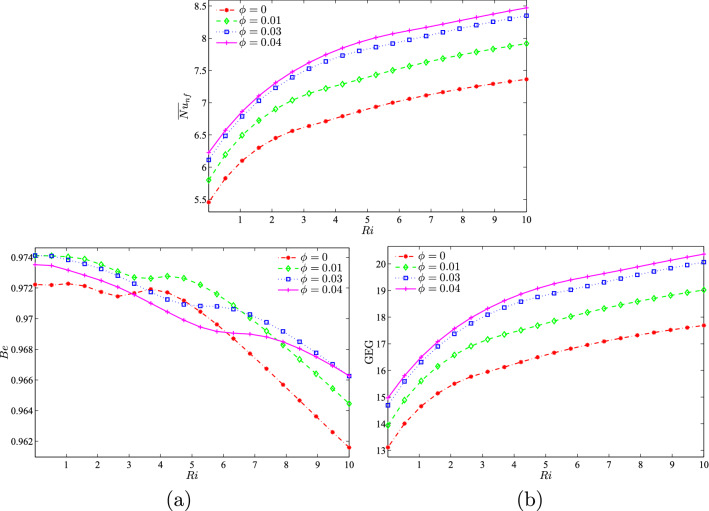
Variation of (**a**) average Nusselt number (${\overline{Nu}}_{nf}$), (**b**) Bejan number (*Be*), and (**c**) global entropy generation (GEG) based on *Ri* for different values of $\phi$ at $Re=100$, $R=0.15$, and *D*1.

Figure 11 illustrates the effect of different nanoparticle volume fractions on the average Nusselt number, Bejan number, and global entropy generation with the Richardson number at $Re = 100$, $R=0.15$, and the location of the inner solid at the centre of the enclosure. As *Ri* increases, the average Nusselt number also increases for all the nanoparticle volume fractions. It was observed that the best performance of the average Nusselt number was at higher nanoparticle volume fractions. Since the value of *Be* was close to 1, the heat transfer irreversibility was dominant as compared to nanofluid irreversibility in the system. The value of *Be* was shown to decreases for all nanoparticle volume fractions. The system is considered to have dominant forced convection if the Richardson number is small. As the system changes to natural convection, the GEG was observed to increase. A higher nanoparticle volume fraction provided the best results for global entropy generation as compared to a smaller nanoparticle volume fraction.

**Figure 12:**
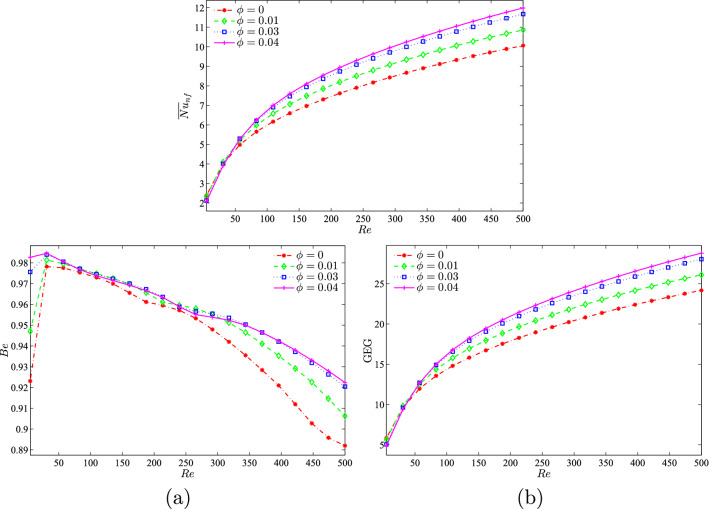
Variation of (**a**) average Nusselt number (${\overline{Nu}}_{nf}$), (**b**) Bejan number (*Be*) and (**c**) the global entropy generation (GEG) with *Re* for different values of $\phi$ at $Ri=1$, $R=0.15$ and *D*1.

Figure 12 depicts the effect of different nanoparticle volume fractions on the average Nusselt number, Bejan number, and the global entropy generation with the Reynolds number at $Ri=1$, $R=0.15$, and the location of the inner solid at the centre of the enclosure. The increase in *Re* is indicative of the increase in the velocity of the top lid. As *Re* increases, the average Nusselt number and GEG increases. The change in the nanoparticle volume fraction also has an effect on the average Nusselt number, Bejan number, and global entropy generation. The best performance for the average Nusselt number and GEG was observed when the nanoparticle volume fraction was at 0.04. From the graph in Fig. 12(a), it was observed that the system was HTI dominant for all nanoparticle volume fractions. As *Re* increased to 50, a corresponding increase was observed in the graph. However, a decrease was observed in the graph when *Re* was greater than 50, possibly due to the combined effects of natural and forced convection.

**Figure 13:**
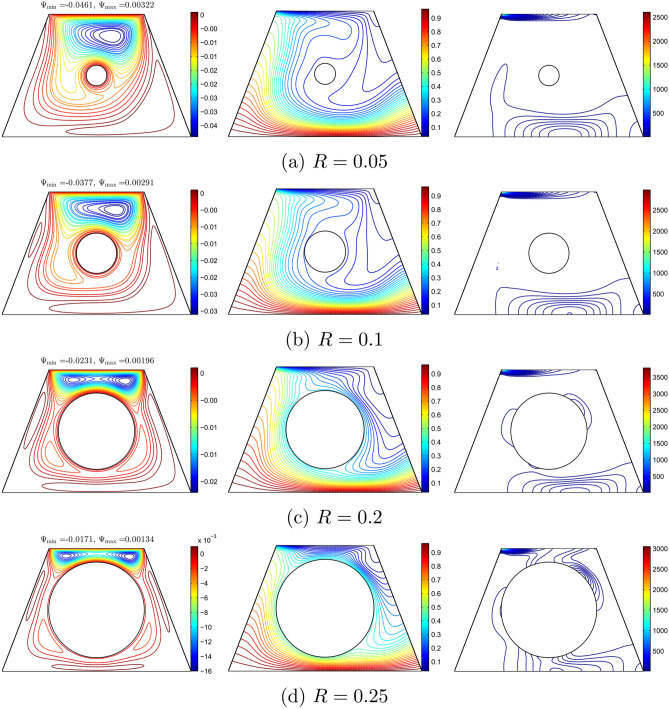
Variation of the streamlines (left), isotherms (middle), and isentropic (right) evolution by solid cylinder radius (*R*) for $Ri=1$, $Re=100$, $\phi =0.02$ and *D*1.

The effects of various radius sizes of the cylinder on the streamlines, isotherms, and isentropic are depicted in Figure 13 with $Ri=1$, $Re=100$, and $\phi =0.02$. As the radius of the cylinder increases, the passage width between the cylinder and the top and bottom wall decreases. Therefore, the size of the cell decreases as the radius of the cylinder increases. It was observed that two cells appeared when the radius of the cylinder was greater than 0.2. As a result, the increase in the solid cylinder showed a high intensity of isotherm lines at the bottom and top of the cavity. For small radius sizes, the intensity of the isentropic was similar to the isentropic lines appearing at the top and bottom of the wall. At a higher radius, the isentropic lines appeared at the solid cylinder as well.

**Figure 14:**
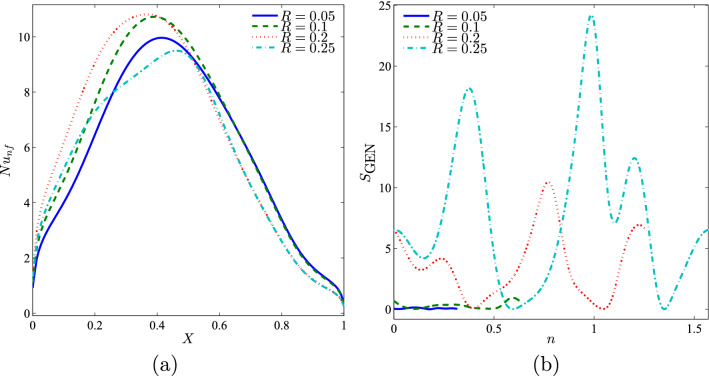
Variation of (**a**) local Nusselt number interfaces with *X* and (**b**) local entropy generation at the outer solid cylinder surface for different *R* at $Ri=1$, $Re=100$, $\phi =0.02$ and *D*1.

Figure 14 depicts the local Nusselt number interfaces with *X* and the local entropy generation at the outer solid cylinder surface with different radius sizes of the solid cylinder. Figure 14(a) shows that the maximum heat transfer occurs mostly at the centre of the cavity. Figure 14(b) illustrates that the local entropy generation is not altered at a smaller radius of the solid cylinder. The local entropy generation depicts a sinusoidal shape for a higher radius of the solid cylinder.

**Figure 15:**
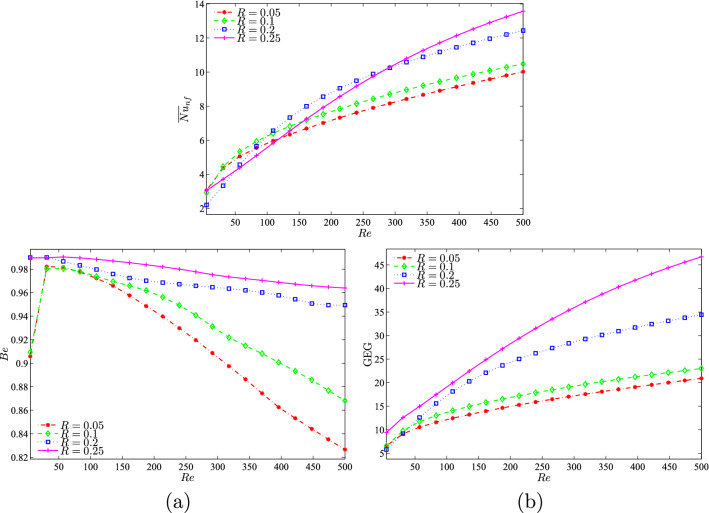
Variation of (**a**) average Nusselt number (${\overline{Nu}}_{nf}$), (**b**) Bejan number (*Be*) and (**c**) the global entropy generation (GEG) with *Re* for different values of *R* at $Ri=1$, $\phi =0.02$ and *D*1.

It was also shown that the radius size changes of the solid cylinder affected the average Nusselt number, Bejan number, and global entropy generation as shown in Figure 15. The average Nusselt number (Figure 15(a)) and global entropy generation (Figure 15(b)) were found to increase when the *Re* number increased. This observation was probably due to the increase in the velocity of the top lid as *Re* increases, thus affecting thermal performance. Furthermore, the Bejan numbers were high for all the radius sizes, thus indicating that the system is HTI dominant as shown in Figure 15(b). The graph illustrates that the Bejan number showed an increasing trend for all radius sizes except for $R=0.25$ up to $Re=50$ and decreased from that point onwards. This is due to the increase in fluid friction as *Re* increases, thus resulting in the decreased Bejan number.

**Figure 16:**
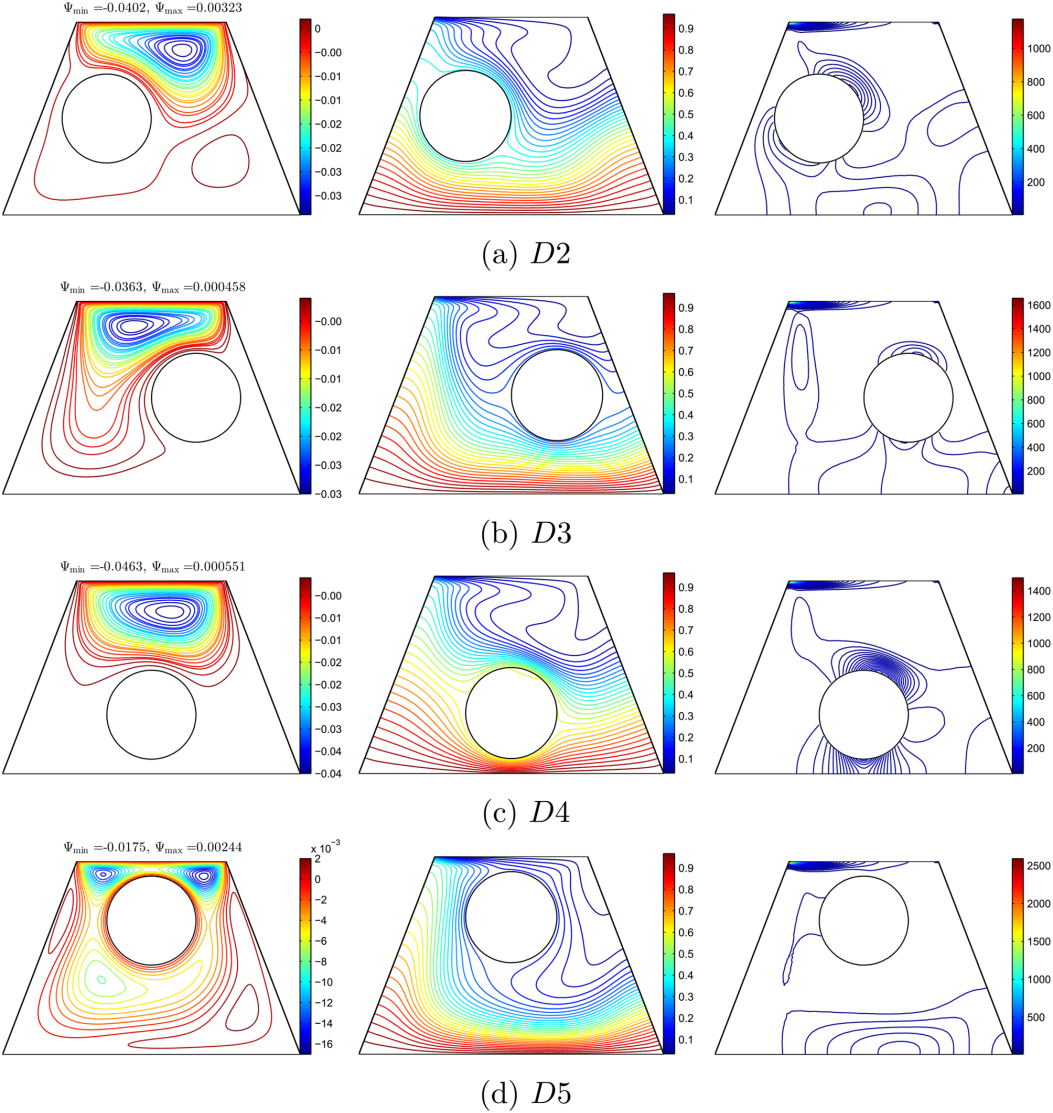
Variation of the streamlines (left), isotherms (middle), and isentropic (right) evolution by location of solid cylinder (*D*) for $Ri=1$, $Re=100$, $\phi =0.02$ and $R=0.15$.

Figure 16 illustrates the effect of various positions of the solid cylinder on the streamlines, isotherm, and isentropic evolution based on the Richardson number, Reynolds number, nanoparticle volume fraction and radius of the solid cylinder with values of 1, 100, 0.02, and 0.15, respectively. In Fig. 16(a) and (b), the position of the solid cylinder was kept constant at $y=0.5$, while the value of *x* was indicated by $x=0.35$ and $x=0.65$ respectively. In Fig. 16(c) and (d) the *x* position was kept constant at 0.5, while the *y* was indicated by $y=0.35$ and $y=0.65$, respectively. It can be observed that the streamlines appeared according to the position of the solid cylinder. However, the streamlines also appeared at the top of the enclosure for all positions of the solid cylinder due to the movement of the top lid wall. In addition, there was no streamline protruding in the solid cylinder. Likewise it can be observed that the isotherm lines appeared in the enclosure in a wavy shape. However, the intensity of the isotherm was lower at the top right of the enclosure for all positions of the solid cylinder. This maybe due to the various temperature distributions and movement of the top lid wall from left to right. However, the isentropic lines showed a high intensity at the top of the enclosure for all positions of the cylinder. It can also be observed that the isentropic lines appeared around the solid cylinder, in high density when the solid cylinder was at the bottom of the enclosure as shown in Figure 16(d).

**Figure 17:**
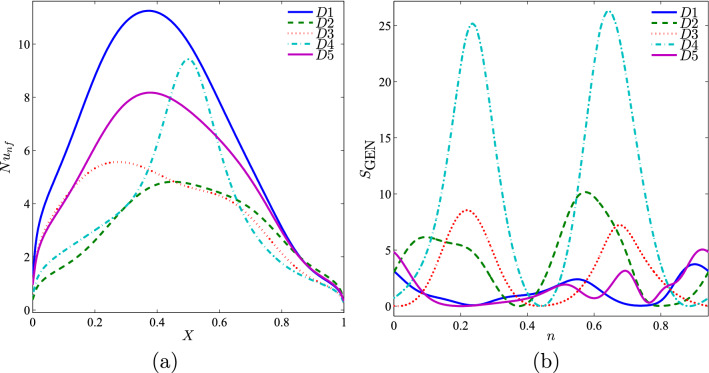
Variation of (**a**) local Nusselt number interfaces with *X* and (**b**) local entropy generation at the outer solid cylinder surface for different *D* at $Ri=1$, $Re=100$, $\phi =0.02$ and $R=0.15$.

Various positions of the solid cylinder based on the local Nusselt number interfaces with *X* and the local entropy generation at the outer solid cylinder surface is depicted in Figure 17. The local Nusselt number was minimum at the vertical position of the cavity wall and maximum between $X=0.2$ and $X=0.6$ based on the position of the solid cylinder. The best thermal performance was obtained when the position of the solid cylinder was at the centre of the enclosure. In addition, the local entropy generation at the outer solid cylinder displayed a sinusoidal shape. It was observed that the local entropy generation was high when the position of the solid cylinder was at the bottom of the enclosure.

**Figure 18:**
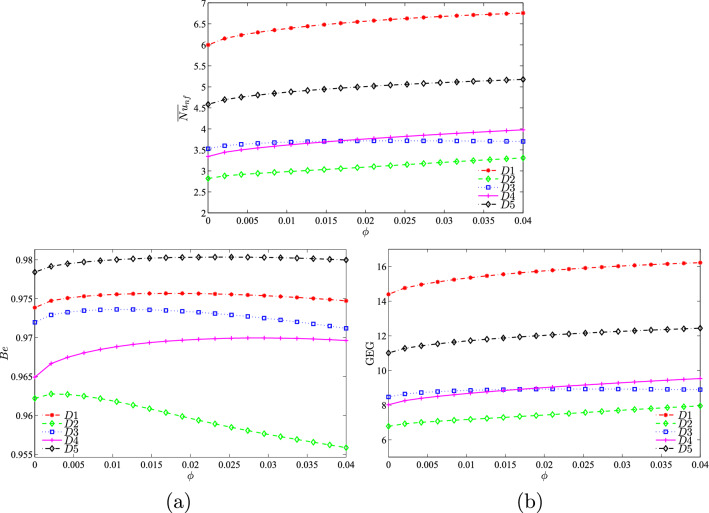
Variation of (**a**) average Nusselt number (${\overline{Nu}}_{nf}$), (**b**) Bejan number (*Be*) and (**c**) the global entropy generation (GEG) with $\phi$ for different values of *D* at $Ri=1$, $Re=1$ and $R=0.15$.

Figure 18 illustrates the average Nusselt number, Bejan number, and global entropy generation with nanoparticle volume fractions for different positions of the solid cylinder at $Ri=1$, $Re=1$, and $R=0.15$. Different solid cylinder positions are thought to affect the average Nusselt number, average entropy generation, and global entropy generation. It can be observed that the average Nusselt number and global entropy generation were high when the position of the solid cylinder was at the centre of the enclosure. However, both the average Nusselt number and global entropy generation increased slightly as the nanoparticle volume fraction increased for all positions except when the solid was located at the left side of the enclosure. The graph in Figure 18(b) shows that the system was HTI dominant for all positions of the solid cylinder since the value of *Be* was greater than 0.5. As the nanoparticle volume fraction number increases, the Bejan number increased slightly for all positions of the solid cylinder except when the solid cylinder was located at the right side of the enclosure.

## Conclusions

In this study, the finite difference method was used to analyse entropy generation and mixed convection in a trapezoidal cavity with various positions of the solid cylinder filled with $\text {Al}_2\text {O}_3$-water nanofluid. The detailed computational results for the streamlines, isotherms, and the local entropy generation for different values of *Ri*, *Re*, nanoparticle volume fractions, radius, and location of the solid cylinder were investigated. Several important conclusions from the study are shown below:

1. The distinct location of the solid cylinder appears to influence flow behaviour, whereby temperature distribution and local entropy generation are both affected by resistance due to the conductive heat transfer in solid cylinder.
2. Increasing the Richardson number and Reynolds number increases the rate of heat transfer as well as nanoparticles volume fraction due to the increment of buoyancy and viscous forces.
3. The maximum average Nusselt numbers are gained for high *R* values, where the average Nusselt number is increased by 30% when *R* is raised from 0 to 0.25.
4. The GEG increases with the rise of *Ri* and *Re*, while a counteractive behaviour is observed for *Be*. The *Be* number also appears to decrease as the *Re* and *Ri* increase.
5. In the case of the intense convection regime (*Re*), the Bejan number is a decreasing function of the solid cylinder radius, whereas the GEG is an increasing function of the solid cylinder radius of the same regime.
6. The heat transfer rate and GEG are high when the solid cylinder is located at the centre of the cavity, while the Bejan number is high when the solid cylinder is located at the top of the cavity.

## References

[1] Roy M, Basak T, Roy S, Pop I (2015). Analysis of entropy generation for mixed convection in a square cavity for various thermal boundary conditions. Numer. Heat Transf. Part A Appl..

[2] Abbasian AA, Mazrouei SS, Mahmoodi M, Ardeshiri A, Aliakbari M (2012). Numerical study of mixed convection flow in a lid-driven cavity with sinusoidal heating on sidewalls using nanofluid. Superlattices Microstruct..

[3] Khorasanizadeh H, Nikfar M, Amanip J (2013). Entropy generation of Cu-water nanofluid mixed convection in a cavity. Eur. J. Mech. B/Fluids.

[4] Sebdani SM, Mahmoodi M, Hashemi SM (2012). Effect of nanofluid variable properties on mixed convection in a square cavity. Int. J. Therm. Sci..

[5] Nayak R, Bhattacharyya S, Pop I (2016). Numerical study on mixed convection and entropy generation of a nanofluid in a lid-driven square enclosure. J. Heat Transf..

[6] Arefmanesh A, Aghaei A, Ehteram H (2016). Mixed convection heat transfer in a CuO–water filled trapezoidal enclosure, effects of various constant and variable properties of the nanofluid. Appl. Math. Model..

[7] Bhattacharya M, Basak T, Oztop HF, Varol Y (2013). Mixed convection and role of multiple solutions in lid-driven trapezoidal enclosures. Int. J. Heat Mass Transf..

[8] Kareem AK, Mohammed H, Hussein AK, Gao S (2016). Numerical investigation of mixed convection heat transfer of nanofluids in a lid-driven trapezoidal cavity. Int. Commun. Heat Mass Transf..

[9] Selimefendigil F, Öztop HF (2018). Modeling and optimization of MHD mixed convection in a lid-driven trapezoidal cavity filled with alumina–water nanofluid: effects of electrical conductivity models. Int. J. Mech. Sci..

[10] Aghaei A, Khorasanizadeh H, Sheikhzadeh G, Abbaszadeh M (2016). Numerical study of magnetic field on mixed convection and entropy generation of nanofluid in a trapezoidal enclosure. J. Magn. Magn. Mater..

[11] Al-Rashed AAAA, Sheikhzadeh GA, Aghaei A, Monfared F, Afrand ASM (2020). Effect of a porous medium on flow and mixed convection heat transfer of nanofluids with variable properties in a trapezoidal enclosure. J. Therm. Anal. Calorim..

[12] Hasib MH, Hossen MS, Saha S (2015). Effect of tilt angle on pure mixed convection flow in trapezoidal cavities filled with water–Al2O3 nanofluid. Procedia Eng..

[13] Chamkha A, Ismael M (2016). Magnetic field effect on mixed convection in lid-driven trapezoidal cavities filled with a Cu–water nanofluid with an aiding or opposing side wall. J. Sci. Eng. Appl..

[14] Javed T, Mehmood Z, Pop I (2017). MHD-mixed convection flow in a lid-driven trapezoidal cavity under uniformly/non-uniformly heated bottom wall. Int. J. Numer. Methods Heat Fluid.

[15] Ababaei A, Abbaszadeh M, Chamkha A, Arefmanesh A (2018). Numerical simulation of double diffusive mixed convection and entropy generation in a lid driven trapezoidal enclosure with a heat source. Numer. Heat Transf. Part A Appl..

[16] Al-Rashed AAAA, Kalidasan K, Kolsi L, Velkennedy R, Aydi A, Hussein AK (2018). Mixed convection and entropy generation in a nanofluid filled cubical open cavity with a central isothermal block. Int. J. Mech. Sci..

[17] Rahimi A, Kasaeipoor A, Malekshah EH, Palizian M, Kolsi L (2018). Lattice Boltzmann numerical method for natural convection and entropy generation in cavity with refrigerant rigid body filled with DWCNTs–water nanofluid-experimental thermo-physical properties. Therm. Sci. Eng. Progr..

[18] Rashidi I, Kolsi L, Ahmadi G, Mahian O, Wongwises S, Abu-Nada E (2020). Three-dimensional modelling of natural convection and entropy generation in a vertical cylinder under heterogeneous heat flux using nanofluids. Int. J. Numer. Methods Heat Fluid Flow.

[19] Alnaqi AA, Hussein AK, Kolsi L, Al-Rashed AAAA, Dong L, Ali HM (2020). Computational study of natural convection and entropy generation in 3-D cavity with active lateral walls. Therm. Sci..

[20] Alsabery A, Tayebi T, Chamkha A, Hashim I (2020). Natural convection of Al2O3–water nanofluid in a non-Darcian wavy porous cavity under the local thermal non-equilibrium condition. Sci. Rep..

[21] Islam T, Alam MN, Asjad MI, Parveen N, Chu YM (2021). Heatline visualization of MHD natural convection heat transfer of nanofluid in a prismatic enclosure. Sci. Rep..

[22] Shah Z, Sheikholeslami M, Kumam P, Shafee A (2020). Modeling of entropy optimization for hybrid nanofluid MHD flow through a porous annulus involving variation of Bejan number. Sci. Rep..

[23] Gibanov NS, Sheremet MA, Oztop HF, Abu-Hamdeh N (2018). Mixed convection with entropy generation of nanofluid in a lid-driven cavity under the effects of a heat-conducting solid wall and vertical temperature gradient. Eur. J. Mech. B/Fluids.

[24] Astanina MS, Sheremet MA, Oztop HF, Abu-Hamdeh N (2018). Mixed convection of Al2O3–water nanofluid in a lid-driven cavity having two porous layers. Int. J. Heat Mass Transf..

[25] Mehmood K, Hussain S, Sagheer M (2017). Mixed convection in alumina–water nanofluid filled lid-driven square cavity with an isothermally heated square blockage inside with magnetic field effect: Introduction. Int. J. Heat Mass Transf..

[26] Goodarzi M, Safaei M, Vafai K, Ahmadi G, Dahari M, Kazi S (2014). Investigation of nanofluid mixed convection in a shallow cavity using a two-phase mixture model. Int. J. Therm. Sci..

[27] Chamkha AJ, Abu-Nada E (2012). Mixed convection flow in single- and double-lid driven square cavities filled with water–Al2O3 nanofluid: Effect of viscosity models. Eur. J. Mech. B/Fluids.

[28] Alinia M, Ganji D, Gorji-Bandpy M (2011). Numerical study of mixed convection in an inclined two sided lid driven cavity filled with nanofluid using two-phase mixture model. Int. Commun. Heat Mass Transf..

[29] Shariat M, Akbarinia A, Nezhad AH, Behzadmehr A, Laur R (2011). Numerical study of two phase laminar mixed convection nanofluid in elliptic ducts. Appl. Therm. Eng..

[30] Alsabery A, Ishak S, Chamkha A, Hashim I (2018). Entropy generation analysis and natural convection in a nanofluid-filled square cavity with a concentric solid insert and different temperature distributions. Entropy.

[31] Alsabery A, Siddheshwar P, Saleh H, Hashim I (2016). Transient free convective heat transfer in nanoliquid-saturated porous square cavity with a concentric solid insert and sinusoidal boundary condition. Superlattices Microstruct..

[32] Mahmoodi M, Sebdani SM (2012). Natural convection in a square cavity containing a nanofluid and an adiabatic square block at the center. Superlattices Microstruct..

[33] Sheremet M, Oztop H, Pop I, Abu-Hamdeh N (2015). Analysis of entropy generation in natural convection of nanofluid inside a square cavity having hot solid block: Tiwari and Das’ model. Entropy.

[34] Alnajem MHS, Alsabery AI, Hashim I (2019). Entropy generation and natural convection in a wavy-wall cavity filled with a nanofluid and containing an inner solid cylinder. IOP Conf. Mater. Sci. Eng..

[35] Alsabery A, Sheremet M, Chamkha A, Hashim I (2019). Impact of nonhomogeneous nanofluid model on transient mixed convection in a double lid-driven wavy cavity involving solid circular cylinder. Int. J. Mech. Sci..

[36] Liao CC, Lin CA (2014). Mixed convection of a heated rotating cylinder in a square enclosure. Int. J. Heat Mass Transf..

[37] Shariat M, Akbarinia A, Nezhad AH, Behzadmehr A, Laur R (2014). MHD mixed convection of nanofluid filled partially heated triangular enclosure with a rotating adiabatic cylinder. J. Taiwan Inst. Chem. Eng..

[38] Billah M, Rahman M, Sharif UM, Rahim N, Saidur R, Hasanuzzaman M (2011). Numerical analysis of fluid flow due to mixed convection in a lid-driven cavity having a heated circular hollow cylinder. Int. Commun. Heat Mass Transf..

[39] Alhashash A (2020). Natural convection of nanoliquid from a cylinder in square porous enclosure using Buongiorno’s two-phase model. Sci. Rep..

[40] Chatterjee D, Gupta SK, Mondal B (2014). Mixed convective transport in a lid-driven cavity containing a nanofluid and a rotating circular cylinder at the center. Int. Commun. Heat Mass Transf..

[41] Alsabery AI, Tayebi T, Chamkha AJ, Hashim I (2018). Effect of rotating solid cylinder on entropy generation and convective heat transfer in a wavy porous cavity heated from below. Int. Commun. Heat Mass Transf..

[42] Alsabery A, Ismael M, Chamkha A, Hashim I (2018). Numerical investigation of mixed convection and entropy generation in a wavy-walled cavity filled with nanofluid and involving a rotating cylinder. Entropy.

[43] Khanafer K, Aithal SM (2013). Laminar mixed convection flow and heat transfer characteristics in a lid driven cavity with a circular cylinder. Int. J. Heat Mass Transf..

[44] Corcione M (2011). Empirical correlating equations for predicting the effective thermal conductivity and dynamic viscosity of nanofluids. Energy Convers. Manag..

[45] Ilis GG, Mobedi M, Sunden B (2008). Effect of aspect ratio on entropy generation in a rectangular cavity with differentially heated vertical walls. Int. Commun. Heat Mass Transf..

[46] Corcione M, Cianfrini M, Quintino A (2013). Two-phase mixture modeling of natural convection of nanofluids with temperature-dependent properties. Int. J. Therm. Sci..

[47] Chon CH, Kihm KD, Lee SP, Choi SU (2005). Empirical correlation finding the role of temperature and particle size for nanofluid (Al2O3) thermal conductivity enhancement. Appl. Phys. Lett..

[48] Ho C, Liu W, Chang Y, Lin C (2010). Natural convection heat transfer of alumina–water nanofluid in vertical square enclosures: An experimental study. Int. J. Therm. Sci..

[49] Bergman TL, Incropera FP (2011). Introduction to Heat Transfer.

